# Interplay of the Mediterranean diet and genetic hypertension risk on blood pressure in European adolescents: Findings from the HELENA study

**DOI:** 10.1007/s00431-024-05435-4

**Published:** 2024-02-13

**Authors:** Gloria Pérez-Gimeno, Miguel Seral-Cortes, Sergio Sabroso-Lasa, Luis Mariano Esteban, Kurt Widhalm, Frederic Gottrand, Peter Stehle, Aline Meirhaeghe, Manon Muntaner, Anthony Kafatos, Angel Gutierrez, Yannis Manios, Costas A. Anastasiou, Marcela Gonzalez-Gross, Christina Breidenassel, Laura Censi, Stefaan de Henauw, Idoia Labayen, Gloria Bueno-Lozano, Azahara I. Rupérez, Luis A. Moreno

**Affiliations:** 1 Growth, Exercise, NUtrition and Development (GENUD), Research Group, Instituto Agroalimentario de Aragón (IA2), Instituto de Investigación Sanitaria Aragón (IIS Aragón) Universidad de Zaragoza, Zaragoza, Spain; 2 Centro de Investigación Biomédica en Red de Fisiopatología de la Obesidad y la Nutrición (CIBERObn), Instituto de Salud Carlos III, Madrid, Spain; 3 Genetic and Molecular Epidemiology Group (GMEG), Spanish National Cancer Research Centre (CNIO), Madrid, Spain; 4 Escuela Politécnica de La Almunia, Universidad de Zaragoza, Zaragoza, Spain; 5 Division of Clinical Nutrition and Prevention, Department of Pediatrics, Medical University of Vienna, 1090 Vienna, Austria; 6 Univ. Lille, Inserm, CHU Lille, - INFINITE - Institute for Translational Research in Inflammation, Lille, France; 7 Department of Nutrition and Food Sciences, University of Bonn, Bonn, Germany; 8 Risk Factors and Molecular Determinants of Aging-Related Diseases (RID-AGE), Centre Hosp. Univ Lille, Institut Pasteur de Lille, Université de Lille, Lille, France; 9 University of Crete School of Medicine, Heraklion, Crete, Greece; 10 University of Granada, Granada, Spain; 11 Institute of Agri-food and Life Sciences, Hellenic Mediterranean University Research Centre, Heraklion, Greece; 12 ImFine Research Group, Department of Health and Human Performance, Facultad de Ciencias de la Actividad Física y del Deporte-INEF, Universidad Politécnica de Madrid, Madrid, Spain; 13 Council for Agricultural Research and Economics, Research Centre for Food and Nutrition, Granada, Italy; 14 Department of Public Health and Primary Care, Faculty of Medicine and Health Sciences, Ghent University, Ghent, Belgium; 15 Department of Health Sciences, Institute for Sustainability & Food Chain Innovation, Public University of Navarra, Pamplona, Spain

**Keywords:** Adolescents, Hypertension, Gen-diet interaction, Mediterranean diet, GRS

## Abstract

Early-life onset of high blood pressure is associated with the development of cardiovascular diseases in adulthood. In adolescents, limited evidence exists regarding the association between adherence to the Mediterranean Diet (MedDiet) and normal blood pressure (BP) levels, as well as its potential to modulate genetic predisposition to HTN. This study investigated the interaction between a MedDiet score and a recently developed HTN-genetic risk score (HTN-GRS) on blood pressure levels in a European adolescent cohort. The MedDiet score was derived from two non-consecutive 24-h dietary recalls and ranged from 0 (indicating low adherence) to 9 (indicating high adherence). Multiple linear regression models, adjusted for covariates, were employed to examine the relationship between the MedDiet score and BP *z*-scores and to assess the interaction effects between the MedDiet score and HTN-GRS on BP z-scores. MedDiet score showed a negative association with *z*-systolic BP (SBP) (*ß* = −0.40, *p* < 0.001) and *z*-diastolic BP (DBP) (*ß* = −0.29, *p* = 0.001). Additionally, a significant interaction effect was identified between the MedDiet score and HTN-GRS on *z*-SBP (*ß* = 0.02, *p* < 0.001) and *z*-DBP (*ß* = 0.02, *p* < 0.001). The modulatory effect of the MedDiet was more pronounced in females than in males, and HTN-GRS exhibited a stronger influence on DBP than on SBP.

*Conclusion*: The study suggests that higher adherence to the MedDiet is associated with reduced BP levels in adolescents and provides evidence of a genetic-diet interaction influencing BP in adolescents.

**Table:** 

**What is Known:** *• Adherence to the Mediterranean diet may reduce BP levels.*	
**What is New:** *• It is the first study to assess the connection between adherence to a Mediterranean diet, a hypertension genetic risk score, and how they interact in influencing blood pressure.* *• It is conducted within a multicenter cohort of European adolescents.*

**Supplementary Information:**

The online version contains supplementary material available at 10.1007/s00431-024-05435-4.

## Introduction

Blood pressure (BP) is the primary modifiable risk factor for cardiovascular diseases (CVD) in adults [1]. In children, the prevalence of hypertension (HTN) has risen from 1.3% in the 1990s to 6% in the 2010s [2]. The most strongly correlated predictive factor linked to HTN trajectory in adulthood is the development of HTN during childhood [3].

During the last decades, adolescents’ dietary behaviors are far from the recommended dietary patterns predecessors [4]. And there has been a significant increase in the consumption of ultra-processed foods representing nearly half of their daily energy intake among European adolescents [5] and 38% among Spanish adolescents [6]. This has been associated with a higher prevalence of HTN [7]. The Mediterranean diet (MedDiet) has been widely studied, due to its protective role in CVD and their main risk factors [8].

In children and adolescents, the literature regarding the association between adherence to MedDiet and BP remains scarce. A recent study in Spanish adolescents reported a combined effect of a high MedDiet adherence together with taking a nap with consistent low BP levels [9]. Moreover, a recent meta-analysis showed that adolescents who followed a healthy dietary pattern decreased their BP levels [10].

Finally, individual responses to a given dietary pattern might vary based on individuals’ genotype [11]. Growing evidence has led to an increased number of studies investigating the interplay between different diets and genotypes [12]. Genetic tools, such as genetic risk scores (GRS), which involve a combination of single nucleotide polymorphisms (SNPs), have been used to estimate the predisposition to certain diseases [13]. In adults, there is evidence of an attenuating effect of MedDiet on the genetic predisposition to cardiometabolic complications, including HTN [14]. However, few studies have assessed the interaction between the combination of SNPs and MedDiet and its impact on BP levels in European adolescents. Another HELENA study observed a lower diastolic BP in male adolescents with high adherence to the MedDiet and fewer risk alleles [15]. Although the evidence between gene-MedDiet relationship in the context of HTN is limited, it is crucial to identify genetic and environmental factors to prevent future cardiovascular events linked to HTN. Therefore, the aim of our study was to assess whether the MedDiet influences or not the effect of an HTN GRS on BP levels through interaction models in European adolescents. We hypothesized that the healthy benefits of MedDiet attenuate the genetic risk of HTN on the high BP levels.

## Materials and methods

### Subjects and study design

Data were collected from a randomly selected cohort of European adolescents (aged 12.5–17.5 years) participating in the Healthy Lifestyle in Europe by Nutrition in Adolescence (HELENA) cross-sectional study. The HELENA study was conducted in 10 European cities (Athens, Greece; Dortmund, Germany; Ghent, Belgium; Heraklion, Crete; Lille, France; Pécs, Hungary; Rome, Italy; Stockholm, Sweden; Vienna, Austria; and Zaragoza, Spain) between 2006 and 2007 [16]. Blood samples were obtained by selecting classes from various districts within each center, with an average of 100–200 adolescents per center. Sample size for blood parameters was estimated considering the highest variability of the planned measurements (Supplementary Fig. 1) [17]. The HELENA study was approved by the human ethics committees of each country involved in the study [18].

### Physical examination

Weight and height were measured by trained researchers in underwear and barefoot with an electronic scale (Type SECA 861) and a stadiometer (Type SECA 225), following a standardized protocol [19], and BMI was calculated from height and weight (kg/m^2^).

A previously validated automated digital BP device for clinical use (OMRON M6 (HEM-7001-E)) [20] was used to measure the systolic and diastolic BP. BP was measured two times in a sitting position with a 10-min interval in between. The cuff size was adapted to the arm circumference of each adolescent, and the lowest recorded BP levels were used. The entire process of BP measurement has been explained in detail previously [21]. For the analysis, the lowest blood pressure (BP) recording was used. Then, standardized *z*-scores for systolic BP (SBP) and diastolic BP (DBP) variables were calculated based on age and sex-specific cut-off points [22]_._ In addition, pubertal status was assessed according to Tanner’s stage by a well-trained physician [23].

### Dietary intake assessment

Two non-consecutive, self-administered 24-h recalls were obtained to determine the adolescents’ dietary intake within a time span of 2 weeks. Each assessment was computerized by a tool previously validated in Flemish adolescents [24]: the HELENA dietary assessment tool (HELENA-DIAT) [25]. HELENA-DIAT allowed participants to select all foods and beverages consumed in six meals (breakfast, morning snacks, lunch, afternoon snacks, dinner, and evening snacks). Additionally, the multiple source method (MSM) was used to calculate individual usual dietary intake, which corrects variability in dietary data between and within individuals [26].

### Mediterranean diet score

A Mediterranean diet score (MDS) was calculated from the sum of scores for 9 food groups and nutrients: fruits, vegetables and nuts, cereals and roots, fish, dairy products, pulses, unsaturated to saturated fat ratio, meat, and alcohol [27]. Then, a scale ranging from 0 to 9 was developed according to the degree of adherence to the traditional MedDiet, which was previously used within the HELENA study [28]. For vegetables and nuts, cereals and roots, fish, dairy products, pulses, and unsaturated to saturated fat ratio, 1 point was assigned when the mentioned groups were above the sex-specific median, while 0 points were assigned to those adolescents with an intake below the sex-specific median. Regarding meat and processed meat, those adolescents with meat consumption below the median were scored with 1 and those above the sex-specific median with 0. Alcohol intake was negatively considered in adolescents. Thus, adolescents with any form of alcohol consumption were assigned 0 points, while those adolescents with no alcohol consumption were assigned 1 point to the MDS [29]. Finally, each food group was computed into a final score, and those adolescents with a higher MDS had higher adherence to MedDiet and vice versa.

### Genomic information

A certified laboratory performed the blood collection, transport, and analysis according to standard methods [30]. The Institute of Nutritional and Food Sciences (IEL) of the University of Bonn performed the blood sampling in EDTA K3 tubes for DNA extraction, collection, and storage. Then, samples were sent to the Centre de Ressources Biologiques (CRB-IPL) (BB-0033-00071 Institut Pasteur de Lille, F-59000 Lille, France) for further analyses. Specifically, DNA was obtained from white cells using the Puregene kit (QIAGEN, Courtaboeuf, France) and was stored at −20 °C until samples were genotyped using the Illumina Global Screening Array chip. After quality control, ~ 600,000 genotyped SNPs were available. Additionally, around 7 million SNPs were obtained with imputation using the Haplotype Reference Consortium reference panel. SNPs were excluded if imputation quality was < 0.3.

### Genetic risk score

A HTN genetic risk score previously developed with HELENA participants [31] was used to analyze the influence of genetic information on the association between MedDiet, adiposity, and cardiometabolic biomarkers. These HTN-GRS were based on 16 single nucleotide polymorphism (SNP) significantly associated with HTN. The main characteristics of the 16 SNPs included in the HTN-GRS are shown in Supplementary Table 1.

### Statistical analysis

To test the variables’ normality, the Shapiro-Wilk test was performed. Since not all variables had a normal distribution, the descriptive characteristics of continuous variables were displayed by the median and interquartile range (IQR). The Mann-Whitney-Wilcoxon test was used to assess differences by sex.

Initially, multiple linear regression models were built for each z-score (SBP and DBP) to assess the association between MDS and BP, considering whether the HTN-GRS score mediated this relationship or not.

Subsequently, these models were adjusted for confounding factors, specifically Tanner’s stage and center: north/center Europe (Dortmund, Gent, Lille, Birmingham, Vienna, Pecs, and Stockholm) and South Europe (Athens, Heraklion, Rome, Zaragoza, and Modena). The *z*-scores for SBP and DBP were utilized as the outcome variables in this analysis. Additionally, multiple linear regression models were constructed separately for each sex. These sex-specific models, which were also adjusted for the Tanner stage and the study center, facilitated a more thorough investigation of the interplay between the MDS-HTN-GRS interaction and BP within each sex group.

All statistical analyses were performed using Rstudio version 1.2.5019 (Rstudio Team (2015). RStudio: Integrated Development for R. RStudio, Inc., Boston, MA, URL http://www.rstudio.com/). The significance level was set at *p* < 0.05.

## Results

### Characteristics of the population

A total of 548 adolescents (53% females) with complete genetic, BP, and dietary information were included in the study (Supplementary Fig. 2). Table 1 shows the main characteristics of the HELENA participants, categorized by sex. Significant differences were observed in height and weight (*p* < 0.001), whereas no significant differences were observed in BMI. Additionally, significant differences (*p* < 0.001) were found in SBP levels and in both SBP and DBP *z*-scores. In terms of diet and genetics, no significant differences were found in the adherence to MDS and HTN-GRS among the participants.

**Table 1:** Demographic, anthropometric, and metabolic characteristics of the adolescents participating in the HELENA study

	**Total**	**Male**	**Female**	***p*-value**
	*n* = 548	*n* = 256	*n* = 292	
**Age (years)**	14.8 (13.8–15.8)	14.8 (13.8–15.8)	14.8 (13.8–15.8)	0.716
**Height (cm)**	166.5 (159.9–172.7)	170.6 (164.5–177.5)	162.9(158.2 –167.8)	**< 0.001**
**Weight (kg)**	59.1 (50.5–65.2)	62.2 (52.1–70.2)	56.5 (49.7–61.6)	**< 0.001**
**BMI (kg/m**^**2**^**)**	21.2 (18.7–22.9)	21.2 (18.5–22.9)	21.2 (18.8–23.0)	0.324
**SBP (mmHg)**	117 (108–124)	121 (112–129)	113 (105–120)	**< 0.001**
**DBP (mmHg)**	64 (59–70)	64 (58–69)	65 (60–70)	0.233
**SBP *z*-score**	0.65 (0.02–1.30)	0.75 (0.12–1.41)	0.57 (–0.07–1.20)	0.121
**DBP *z*-score**	0.63 (–0.1–1.2)	1.27 (0.6–1.7)	0.08 (–0.6–0.8)	**< 0.001**
**MDS**	4.3 (3–5)	4.3 (3–5)	4.3 (3–5)	0.504
**HTN-GRS**	16 (15–18)	16 (15–17)	16 (15–18)	0.349

Mann-Whitney-Wilcoxon test was performed to assess differences between sex in non-normal variables. Values of these variables are presented as median (p.25–p.75)

*SBP *systolic blood pressure, *DBP* diastolic blood pressure, *MDS* Mediterranean diet score, resulting from the sum of nine food subgroups, *HTN-GRS* hypertension-genetic risk score, resulting from the sum of risk alleles of HELENA participant

### Interaction between MedDiet score and HTN-GRS on BP variables

Table 2 shows the associations between MDS and both, SBP and DBP, *z*-scores, mediated by the interaction between HTN-GRS and MDS. A general linear model, which included the Tanner stage and center as covariates, showed that for each point increase in the MDS, there was a protective effect observed in both *z*-SBP (*ß* = −0.4) and *z*-DBP (*ß* = −0.29).

**Table 2:** Multiple linear regression models of the Mediterranean diet (MD) effect and MD and hypertension-related genetic risk score (HTN-GRS) interaction on SBP and DBP *z*-scores

	**MDS**		**HTN-GRS:MDS**	
	Beta	*p*-value	Beta	*p*-value
***All participants***				
SBP	−0.40	< 0.001	0.02	< 0.001
DBP	−0.29	< 0.001	0.02	< 0.001
***Male***				
SBP	−0.57	< 0.001	0.03	< 0.001
DBP	−0.37	0.001	0.03	< 0.001
***Female***				
SBP	−0.32	0.003	0.01	0.022
DBP	−0.44	< 0.001	0.02	< 0.001

Adjusted by the Tanner and center (north/center Europe (Dortmund, Gent, Lille, Birmingham, Vienna, Pecs, and Stockholm) and South Europe (Athens, Heraklion, Rome, Zaragoza, and Modena)

*SBP* systolic blood pressure, *DBP* diastolic blood pressure, *MDS* Mediterranean diet score, resulting from the sum of nine food subgroups, *HTN-GRS* hypertension-genetic risk score, resulting from the sum of risk alleles of HELENA participants

Additionally, when holding MDS constant, an increase of one point in the HTN-GRS is associated with an increase in the *z*-scores of BP in both cases for *z*-SBP (*ß* = 0.02) and *z*-DBP (*ß* = 0.02). Due to significant differences in sex (Table 1) for BP *z*-scores, separate models were constructed for each sex (Table 2). In males, MDS exhibited a stronger protective effect on SBP compared to DBP, *z*-SBP (*ß* = −0.57), and *z*-DBP (*ß* = −0.37). In contrast, in females, the MDS demonstrated a higher protective role for DBP than for SBP, *z*-SBP (*ß* = −0.44), and *z*-DBP (*ß* = −0.34).

**Fig. 1:**
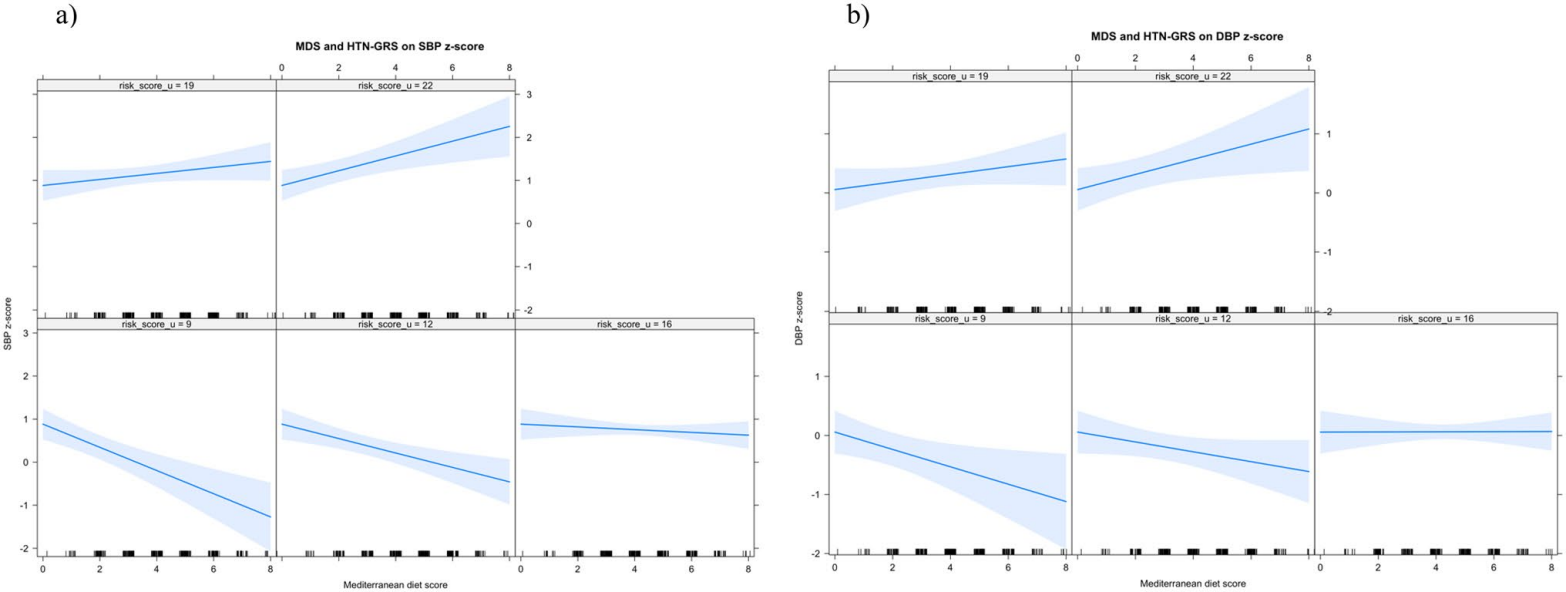
Interaction models between the Mediterranean diet score and hypertension genetic score on SBP *z*-score (**a**) and DBP *z*-score (**b**) in males. Different boxes have been outlined, each linked to a varying number of risk alleles. As the count of risk alleles rises (from bottom to top), the gradient of the Mediterranean diet score undergoes a transformation from a negative slope, indicating a protective impact at 9 and 12 risk alleles, to a slightly diminished effect at 16 risk alleles, eventually culminating in a positive gradient, where 19 and 22 risk alleles serve as risk factors. Abbreviations: DBP z-score, diastolic blood pressure *z*-score; HTN-GRS, hypertension-genetic risk score; MDS, Mediterranean diet score; SBP z-score, systolic blood pressure *z*-score

**Fig. 2:**
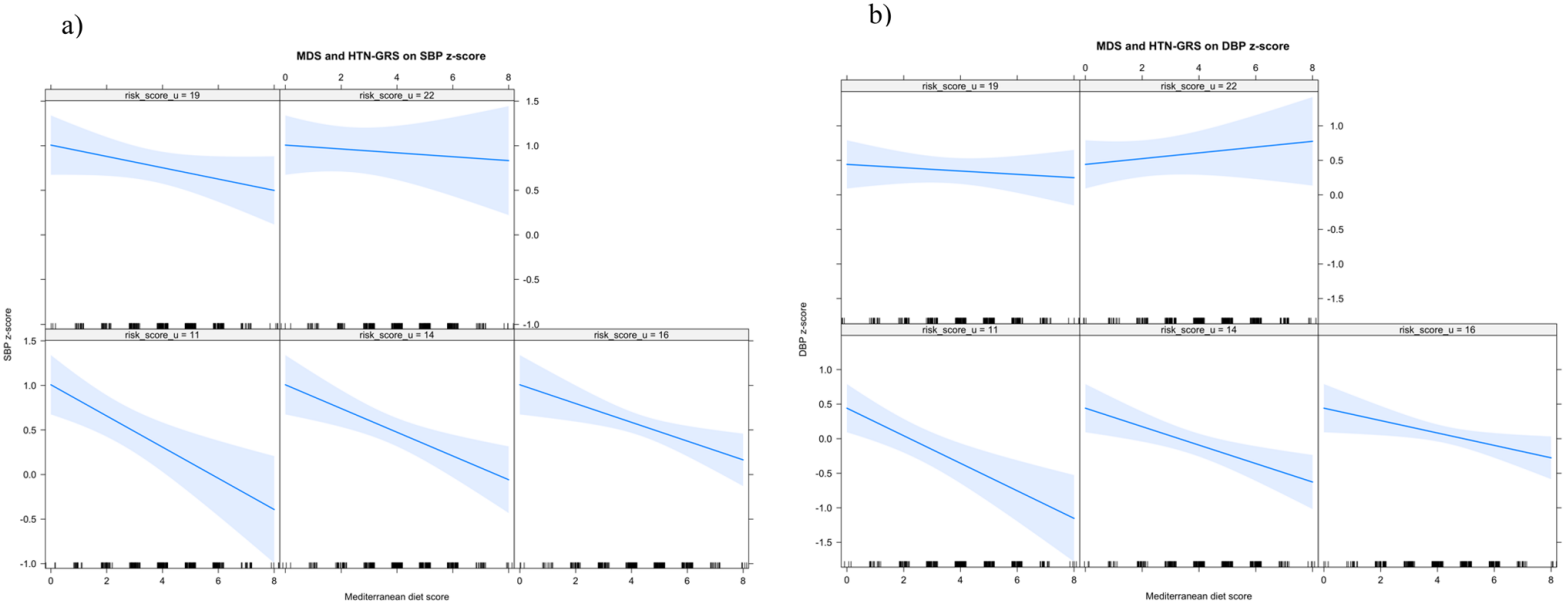
Interaction models between the Mediterranean diet score and hypertension genetic score on SBP *z*-score (**a**) and DBP *z*-score (**b**) in females. Different boxes have been outlined, each linked to a varying number of risk alleles. As the count of risk alleles rises (from bottom to top), the gradient of the Mediterranean diet score on SBP consistently displays a negative slope, indicating a protective role across all cases. However, for DBP, individuals with the highest number of risk alleles (22) exhibit a slight positive gradient in the Mediterranean diet score, suggesting a potential risk factor. Abbreviations: DBP *z*-score, diastolic blood pressure *z*-score; HTN-GRS, hypertension-genetic risk score; MDS, Mediterranean diet score; SBP *z*-score, systolic blood pressure *z*-score

Consistent with the global model, the HTN-GRS was found to modify the association between MDS and BP, with a more pronounced impact observed in males compared to females, as depicted in Figs. 1 and 2. These figures show the interaction effects of the HTN-GRS and MDS on SBP and DBP in males and females, respectively. Adherence to MedDiet showed a protective role in participants with a lower number of risk alleles. In females with fewer than 22 risk alleles, MedDiet showed a protective role against the higher BP values, whereas for males, the effect was less pronounced, showing a protective role for those with fewer than 16 risk alleles. Furthermore, it was observed that in both males and females, HTN-GRS exerted a stronger influence on DBP than on SBP.

## Discussion

In this study, it was observed that the adherence to MedDiet modulates the risk of HTN, showing a protective influence on both SBP and DBP *z*-scores. It is noteworthy that the protective effect of MedDiet adherence diminishes as the number of risk alleles contained in the HTN-GRS increases. Furthermore, this effect is higher in females than in males.

To our knowledge, this is the first study to assess the interaction effects of MDS and a HTN-related GRS among European adolescents. Another study in European adolescents found a modulatory effect of obesity risk alleles on DBP [15] using a specific GRS for obesity rather than for HTN. Our study also observed a modulatory effect of MedDiet on HTN risk alleles for both SBP and DBP in both sexes, whereas the abovementioned study [15] showed impact on DBP only in males.

In the context of the association between adherence to MedDiet and BP, our findings align with those of previous research. A study (*n* = 1378) conducted on a population with similar characteristics to HELENA participants showed a lower SBP (*ß* = −2.60 mmHg; 95% CI: −5.18–0.02) and DBP (*ß* = −1.65 mmHg; 95% CI: −4.00–0.71) among adolescents with high adherence to MedDiet and frequent siestas compared with adolescents with low adherence to MedDiet and no siestas [9]. However, it is important to note that only the reduction in SBP reached statistical significance. In contrast to Mesas et al., in the study, both SBP and DBP were significantly associated with MDS, but the nap variable was not examined. Additionally, a meta-analysis by Cowell et al. showed the beneficial effects of MedDiet on BP reduction in adults [32]. However, literature on gene-diet interactions, specifically focusing on MedDiet and HTN, remains scarce [33]. As well as assessing the total impact of MedDiet as a dietary pattern on individuals´ genotype, other studies have assessed the effect of specific foods, micronutrients, or macronutrients. For instance, one study identified a gene-diet interaction between s699-*AGT* and rs1799722-*BDKRB2* and the consumption of three micronutrients (sodium, magnesium, and calcium) on BP variation in adults [34]. Another study observed that the rs1799998-*CYP11B2* affects the risk of HTN in Japanese men and high-salt intake levels strengthen this association [35]. Similarly, an intervention study examined the interaction effects of the consumption of a high-saturated-fat diet and rs4343-*ACE* on BP and found that GG carriers had a higher SBP than AA/AG carriers in response to the intervention [36].

The diversity in different methodologies to assess diet among studies (through nutrients, foods, or dietary patterns; and by 24-h recalls, food frequency questionnaires) contribute to the complexity of gene-diet interactions assessment and implementing tailored interventions [37]. In addition to the adherence to the MedDiet, adherence to other healthy dietary patterns, such as the dietary approaches to stop hypertension (DASH) diet, has been found to have a protective effect on BP [38].

In a study conducted on 1068 children aged 5 to 7 years old, adherence to the DASH diet and a low GRS risk profile for HTN were associated with lower BP levels [39]. Another study in adults found that homozygotes for the G allele of the angiotensinogen genotype (G-6A *ANG* polymorphism) showed a lower BP decrease during DASH diet intervention [40].

The differences between DASH and MedDiet are mainly due to recommendations regarding fat consumption. MedDiet emphasizes the consumption of healthy unsaturated and omega-3 fats found in foods such as extra virgin olive oil and fish [41]. Conversely, DASH has more stringent limitations on saturated fat intake [38]. Additionally, because of the origins of the DASH diet, sodium intake has become an important consideration, whereas sodium is not the main focus on the MedDiet.

Several meta-analyses have compared different dietary patterns in adult studies, showing that the DASH diet has more evidence and greater effects on BP reduction than MedDiet [42]. However, in the HELENA study, sodium intake was not recorded, so we have not been able to assess the adherence to the DASH characteristics. Furthermore, the MedDiet score used in the HELENA study was previously validated, providing confidence in the methodology employed for its assessment [29].

A HTN-related GRS, based on a combination of 16 SNPs associated with SBP and DBP in adolescents, was used in the present study [31]. Other studies have used GRS developed from adult populations [39, 43–45] and attempted to establish associations with SBP or DBP in cohorts of children and/or adolescents. Several of these studies used the same adult HTN-GRS, but different results were obtained. Some showed associations between the GRS and DBP [39], while other studies showed an association with SBP [44]. Similarly, to the present study, a GRS based on 13 SNPs was associated with both SBP and DBP. In that study, individuals with higher GRS scores had nearly double the risk of developing HTN in adulthood (*OR* = 1.82, 95% CI = 1.53–2.16) [45]. Inconsistent findings from different studies highlight the importance of standardizing studies and using GRS developed within populations with similar ethnic characteristics to the population of interest. Conducting meta-analyses of gene-diet interactions in randomized intervention trials and prospective cohorts that consider similar dietary interventions and genetic markers is essential to advance our understanding of these complex interactions [14].

The present study exhibits some strengths to consider: the HELENA study comprises a large cohort sample and the results obtained can be extrapolated to adolescents with Caucasian ancestors. Second, dietary data were collected using a validated tool, and food group consumption was assessed using a standardized adherence scale. Third, we used an unweighted HTN-GRS aligned with the recent updated guidelines of the American Academy of Pediatrics for screening and management of high BP in children and adolescents [46]. Finally, this study is focused on adolescents and BP individually, in contrast to many other studies that are focus on adults and have assessed multiple components of metabolic syndrome or various cardiovascular risk factors.

On the other hand, limitations of this study should also be recognized. First, no cause-effect relationship can be drawn due to the characteristics of the cross-sectional study design. Second, there is a lack of repeated BP measurements over time to accurately classify the participants according to their BP levels. Furthermore, an oscillometric device was used for BP measurement, which often resulted in lower BP values [2].

While this study underscores the significant role that genetics play in BP, it is crucial to avoid over-attributing every disease-related phenotype to one’s genetic predisposition, thereby neglecting the importance of other health and lifestyle-related factors. Public health policies should promote the MedDiet, given that it not only provides benefits for BP, but it has also been associated with higher health-related quality of life [47].

## Conclusions

Adherence to the MedDiet modulates the deleterious effect of the GRS on BP values in adolescents. The interplay between genes and diet exhibited a more pronounced impact on HTN in males compared to females. These findings shed light on the connection between genetic predisposition, dietary choices, and the potential for HTN development, suggesting the need for sex-specific considerations in preventive strategies.

### Supplementary Information

Below is the link to the electronic supplementary material.

Supplementary file1 (DOC 280 KB)

## Data Availability

The data is not publicly accessible due to ethical constraints aimed at safeguarding patient confidentiality. However, the data that support the findings of this study and the data sets analyzed in the present study can be obtained upon a reasonable request to the corresponding author.

## Abbreviations

BP: Blood pressure
DBP: Diastolic blood pressure
GRS: Genetic Risk Score
HELENA: Healthy Lifestyle in European by Nutrition in Adolescence
HTN: Hypertension
MedDiet: Mediterranean Diet
MDS: Mediterranean diet score
SBP: Systolic blood pressure
