# Supplementary material for: Interplay of the Mediterranean diet and genetic hypertension risk on blood pressure in European adolescents: Findings from the HELENA study
Source: Eur J Pediatr. 2024 Feb 13;183(5):2101–10. doi: 10.1007/s00431-024-05435-4 (PMC11035432; doi:10.1007/s00431-024-05435-4)
Supplement: Supplementary file 1 — Supplementary file1 (DOC 280 KB) [file 431_2024_5435_MOESM1_ESM.doc]

# Supplementary lnformation

**Fig. 1** Sampling procedure schemes. (1) Considering geographic balance and the presence of an experienced research group. (2) Strata by age, sex and school are in every city. (3) Strata by age, sex and school are in every city


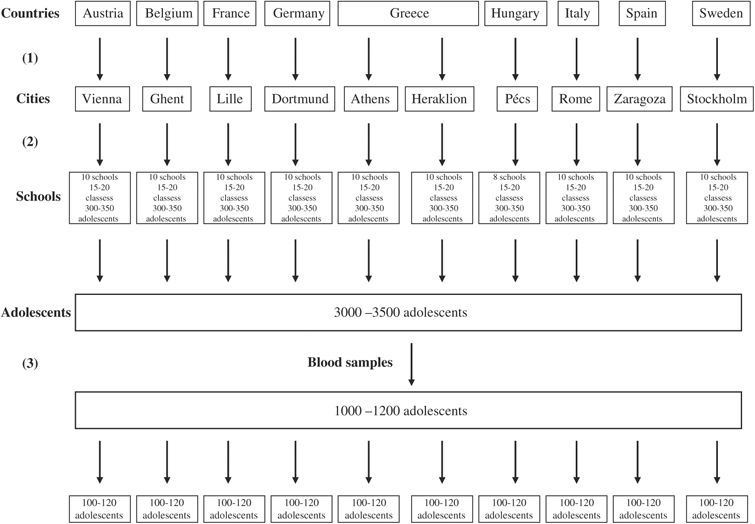


**Fig. 2** Flow chart of the sample selection process


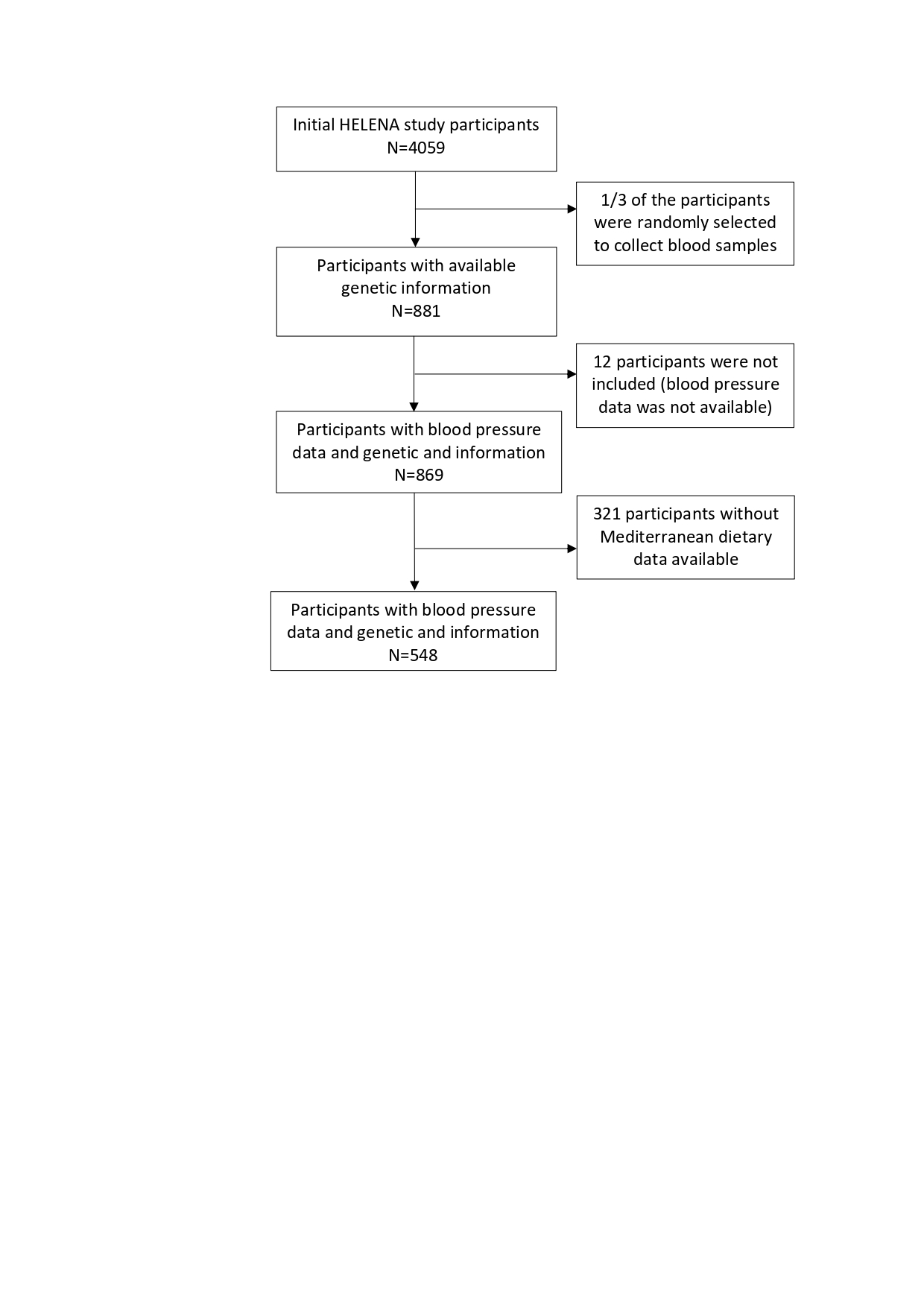


**Supplementary table 1** Main characteristics of the 16 single nucleotide polymorphisms included in the hypertension genetic risk score

| **rs code** | **Nearest gene** | **Alleles (major/minor)** | **MAF** | **Imputation score** | **HWE** |
| --- | --- | --- | --- | --- | --- |
| **rs6433023** | *STK39* | T/C | 0.249 | 0.98477 | 0.069 |
| **rs4580521** | *ULK4* | A/C | 0.346 | 0.99459 | 0.1 |
| **rs4973982** | *ULK4* | G/C | 0.101 | 0.99422 | 0.353 |
| **rs17108817** | *ADRB2* | T/C | 0.488 | 0.94183 | 0.541 |
| **rs76973157** | *SMARCA2* | A/C | 0.117 | 0.99783 | 0.870 |
| **rs7048826** | *SMARCA2* | G/C | 0.13 | 0.99179 | 0.881 |
| **rs10965093** | *SMARCA2* | C/G | 0.433 | 0.9665 | 0.407 |
| **rs62533676** | *PAX5* | T/G | 0.25 | 0.97257 | 0.415 |
| **rs76466243** | *CACNB2* | C/G | 0.138 | 0.99581 | 0.568 |
| **rs75351046** | *PLEKHA7* | C/T | 0.244 | 0.9783 | 0.927 |
| **rs72865722** | *PLEKHA7* | G/A | 0.129 | 0.9939 | 0.880 |
| **rs10832706** | *PLEKHA7* | C/T | 0.269 | 0.99972 | 0.684 |
| **rs17320635** | *ITGA11* | A/G | 0.178 | 0.99943 | 0.487 |
| **rs895135** | *ITGA11* | G/T | 0.361 | 0.92715 | 0.339 |
| **rs8057044** | *FTO* | G/A | 0.465 | 0.99549 | 0.453 |
| **rs113087295** | *UMODL1* | C/T | 0.115 | 0.99787 | 0.316 |
